# Novel Strategy of Using Methyl Esters as Slow Release Methanol Source during Lipase Expression by mut^+^ *Pichia pastoris* X33

**DOI:** 10.1371/journal.pone.0104272

**Published:** 2014-08-29

**Authors:** Arti Kumari, Rani Gupta

**Affiliations:** Department of Microbiology, University of Delhi South Campus, New Delhi, India; Nazarbayev University, Kazakhstan

## Abstract

One of the major issues with heterologous production of proteins in *Pichia pastoris* X33 under AOX1 promoter is repeated methanol induction. To obviate repeated methanol induction, methyl esters were used as a slow release source of methanol in lipase expressing mut^+^ recombinant. Experimental design was based on the strategy that in presence of lipase, methyl esters can be hydrolysed to release their products as methanol and fatty acid. Hence, upon break down of methyl esters by lipase, first methanol will be used as a carbon source and inducer. Then *P. pastoris* can switch over to fatty acid as a carbon source for multiplication and biomass maintenance till further induction by methyl esters. We validated this strategy using recombinant *P. pastoris* expressing Lip A, Lip C from *Trichosporon asahii* and Lip11 from *Yarrowia lipolytica*. We found that the optimum lipase yield under repeated methanol induction after 120 h was 32866 U/L, 28271 U/L and 21978 U/L for Lip C, Lip A and Lip 11 respectively. In addition, we found that a single dose of methyl ester supported higher production than repeated methanol induction. Among various methyl esters tested, methyl oleate (0.5%) caused 1.2 fold higher yield for LipA and LipC and 1.4 fold for Lip11 after 120 h of induction. Sequential utilization of methanol and oleic acid by *P. pastoris* was observed and was supported by differential peroxisome proliferation studies by transmission electron microscopy. Our study identifies a novel strategy of using methyl esters as slow release methanol source during lipase expression.

## Introduction

*Pichia pastoris* is a methylotrophic yeast that is considered as an excellent expression system for heterologous protein production [1]. It has many advantages over *E. coli* and other yeast systems such as better protein secretion efficiency, higher biomass yield and the presence of a tightly regulated methanol inducible promoter alcohol oxidase 1 (p*AOX1*) [1]. However, repeated methanol induction is tedious and methanol evaporates rapidly that can reduce the recombinant protein production. Therefore, the major challenge is to introduce a system that allows slow and continuous release of methanol for steady production of recombinant protein, without the need of repeated methanol induction.

To overcome this problem, we proposed a strategy for lipase producing recombinant mut^+^ *P. pastoris*, with a single methanol induction to release small amount of recombinant lipase, followed by induction with methyl ester. We predicted that recombinant lipase hydrolyses methyl esters into methanol and fatty acid. Methanol released during hydrolysis can induce p*AOX1* to enhance lipase production, whereas fatty acid can be used by *P. pastoris* as a carbon source to maintain the biomass.

In the present study, we validated the proposed strategy using recombinant mut^+^ *P. pastoris* expressing, Lip A, Lip C from *Trichosporon asahii* MSR54 and Lip11 from *Yarrowia lipolytica*.

## Materials and Methods

### Materials

Restriction enzymes were purchased from New England Biolabs (NEB), USA. Taq polymerase and T4 DNA ligase were purchased from Bangalore Genei, India. Gel extraction kit and plasmid isolation kit were purchased from Qiagen, India. Recombinant yeast strain *P. pastoris* X-33 harbouring Lip11 gene from *Yarrowia lipolytica* was taken from the laboratory culture collection. This strain has been submitted to Microbial Type Culture Collection (MTCC) with MTCC number 9517. Zeocine was from Invitrogen. The triacylglycerides, *p*-np esters used in the experiments were procured from Sigma Aldrich. Luria bertani, tryptone, yeast extract, yeast nitrogen base and methanol were purchased from Hi-Media. Sodium chloride was taken from *Sisco Research Laboratories Pvt. Ltd. India* (SRL). Glycosylation kit was procured from G Bioscience (USA).

### Lipase assay and protein estimation

Enzyme assay was performed using *p*-Nitrophenyl palmitate [10] and confirmed by titrimetry [11] using 10% (v/v) olive oil as substrate. One unit of lipase was defined as the amount of enzyme required to release 1 µmole of *p*-nitrophenol or fatty acid respectively, per ml per min at the optimum pH and temperature. Total protein was estimated by the Bradford method as standard protein.

### Cell density measurement

One ml cell culture was pelleted at 5000 g at 10°C, washed and resuspended in 10 mM phosphate buffer saline (PBS) to measure the optical density at 600 nm using UV-1700 pharmaspec spectrophotometer from SHIMANDZU. The dry cell weight was determined after drying 1 ml pelleted culture at 70°C for 24 h and dry cell weight (DCW) was determined gravimetrically.

### Production optimization

Initial cell density in buffered methanol-complex medium (BMMY) was varied from OD_600_ = 2, 4, 6, 8 with 0.5% methanol feeding in 3 h old culture followed by induction after 24 h. Further different methanol concentration *viz*; 0.5%, 1%, 2%, 4%, each was used for induction keeping initial cell density constant in BMMY medium. Methanol induction timing was same as used to optimize initial cell density. These conditions were optimized in 250 ml flask and culture was incubated at 30°C and 200 rpm, over a period of 48 h and lipase activity and biomass was determined as described earlier.

### Process parameter optimization by substituting methyl esters in place of methanol

Various methyl esters *viz*. methyl caprylate, methyl laurate, methyl palmitate, methyl oleate and methyl linoleate were used at the concentration of 0.1% to replace methanol. Cells were grown at 30°C, 200 rpm and first induced with 0.5% methanol after 3 h, followed by induction with different methyl esters (0.1%) after 24 h. Subsequently, the concentration of best methyl ester was standardized by using different concentrations ranging from 0.05% to 0.5% for a period of 120 h.

### Time kinetics of lipase production in optimized conditions

Lipase production was carried out with initial cell density O.D_600_ = 4 and first induction with 0.5% of methanol after 3 h followed by second induction by 2% methanol after every 24 h or 0.5% methyl oleate after 24 h. Lipase activity, protein concentration and cell biomass was analyzed after regular interval of time period till 120 h.

### Measuring concentration of methyl esters and its bi-products

Concentration of methyl oleate and oleic acid was monitored by gas chromatography. Following conditions were used in stabil wax ® - DA column; Temperature – 250°C, Injection mode – split, pressure – 126.6 Kpa, total flow – 149.4 ml/min, column flow – 2.87 ml/min, linear flow – 50.9 cm/sec, purge flow – 3.0 ml/min, split ratio – 50.0 [5].

### TEM analysis and fed batch strategy with methyl oleate as inducer

Fed batch strategy was developed after monitoring the concentration of methyl oleate consumption and 0.1% of methyl oleate was added to the medium after 72 h and results were compared after 120 h. TEM analysis was performed according to Wriessnegger et al., 2007 [7].

### Statistical analysis

All experiments were repeated three times in duplicate. Data was plotted with mean ± SD. Mean and SD was calculated using sigma software.

## Result and Discussion

To substantiate the projected strategy, experimentation were performed on mut^+^ *P. pastoris* expressing different lipases *viz*. Lip A, Lip C from *T. asahii* MSR54 and Lip11 from *Y. lipolytica*. These clones were previously developed in the laboratory (please provide a reference). In the beginning, lipase production was optimised using conventional method of repeated methanol approach, followed by the validation of planned strategy.

### Optimisation of lipase over expression using methanol as inducer

**Figure 1:**
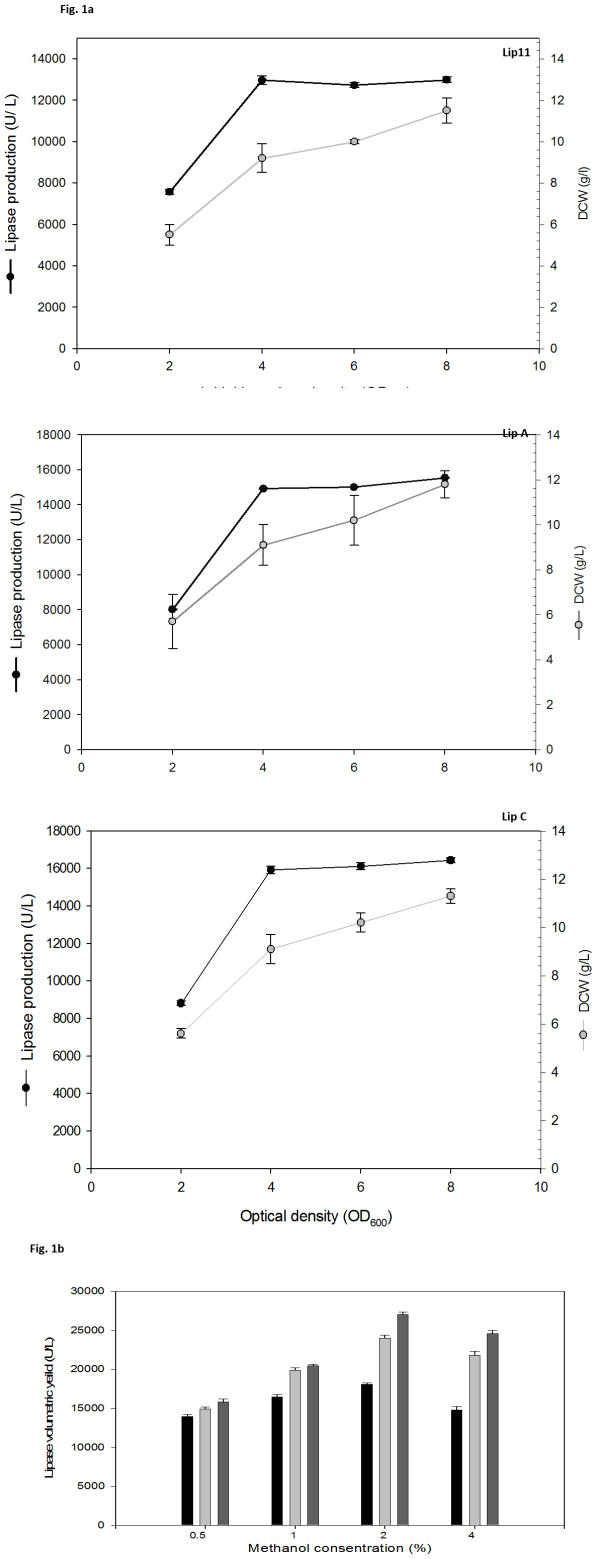
Lipase production as a function of initial O.D (a), and methanol concentration (b) in BMMY medium after 48 h culture at 30°C, 200 rpm. (a) Initial inoculum density was optimized with 0.5% methanol as inducer at 3 h followed by 24 h. Lipase yield (U/L) and DCW (g/l) were calculated after 48 h for Lip 11, Lip B and Lip C. In figure (b), methanol concentration was optimized at initial O.D = 4.0 in BMMY medium.

Initial cell density in BMMY and methanol concentration are the two important factors responsible for lipase over-production in recombinant *P. pastoris* [2]. We observed that there was a linear increase in lipase production of all the lipases from initial O.D_600_ 2 to 4 that became constant beyond OD_600_ 6. Lipase productivity of Lip A and Lip C at OD_600_ was 14190 U/L and 15919 U/L respectively, which later became constant to 14929 for Lip A and 16012 U/L for Lip C at O.D_600_ = 8 (Figure 1), while biomass increased as the O.D increased from 2 to 8. This is in agreement with the previous report of YlLip2 where, high cell density led to decrease in lipase productivity because of lower cell viability [3]. Our analysis suggested that cell density at O.D_600_ = 4 is optimum for the lipase production.

Furthermore, we optimized methanol concentration using initial cell density as O.D_600_ = 4. We found that the rise in methanol concentration from 0.5% to 2% increases lipase volumetric yield of Lip 11 by 1.4 fold to 18070 U/L, Lip A and Lip B by 1.7 fold to 24011 U/L and 27011 U/L, respectively, after 48 h (Figure 1b). Our results indicate that in all the recombinant strains of *P. pastoris* X33, lipase production was improved with an increase in methanol concentration till 2% and declined when methanol concentration reached to 4%. The decrease in lipase production at higher methanol concentration may be due to its adverse effect on cell viability [4]. Hence, we used 2% of methanol concentration for the production of lipases in subsequent experiments.

**Figure 2:**
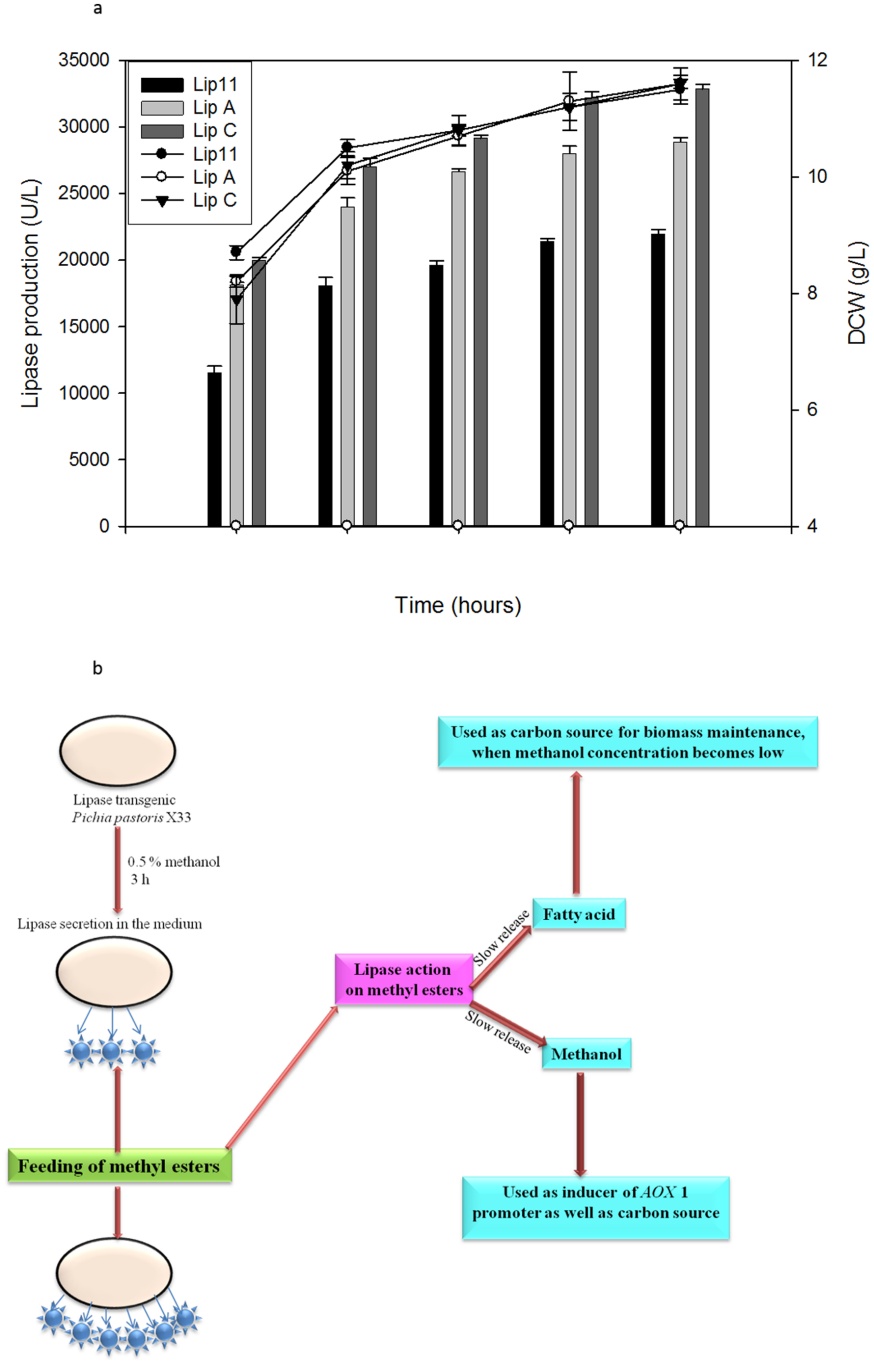
Time profiling of lipase production under optimized conditions using 2% methanol as inducer monitored after every 24 h (A) and schematic representation of proposed hypothesis (B).

We initiated a time course study to investigate lipase production under optimised conditions (initial cell density O.D_600_ = 4 in BMMY medium and methanol concentration 2%) for 120 h. The culture was induced with 2% methanol after every 24 h. Under optimised conditions, we noticed a sharp increase in lipase production and dry cell weight (DCW) for 48 h (Figure 2). However, repeated methanol induction after every 24 h is tedious because methanol evaporates rapidly under small scale culture conditions and it is difficult to maintain constant methanol concentration [3]. Therefore, a gradual process is needed that allows slow and constant release of methanol. The strategy is depicted in figure 2b that shows the use of methyl ester as a source of slow methanol release in lipase expressing recombinants. This technique requires induction by 0.5% methanol after 3 h, followed by postliminary induction with methyl esters. We predicted that the induction with 0.5% methanol in early hours would induce p*AOX*1 to release recombinant lipase and convert it into lipase expressing strain. Subsequently, methyl esters will be hydrolysed to methanol and fatty acids, where methanol could sustain the production of lipase by constantly inducing p*AOX*1.

### Selection of methyl esters

**Figure 3:**
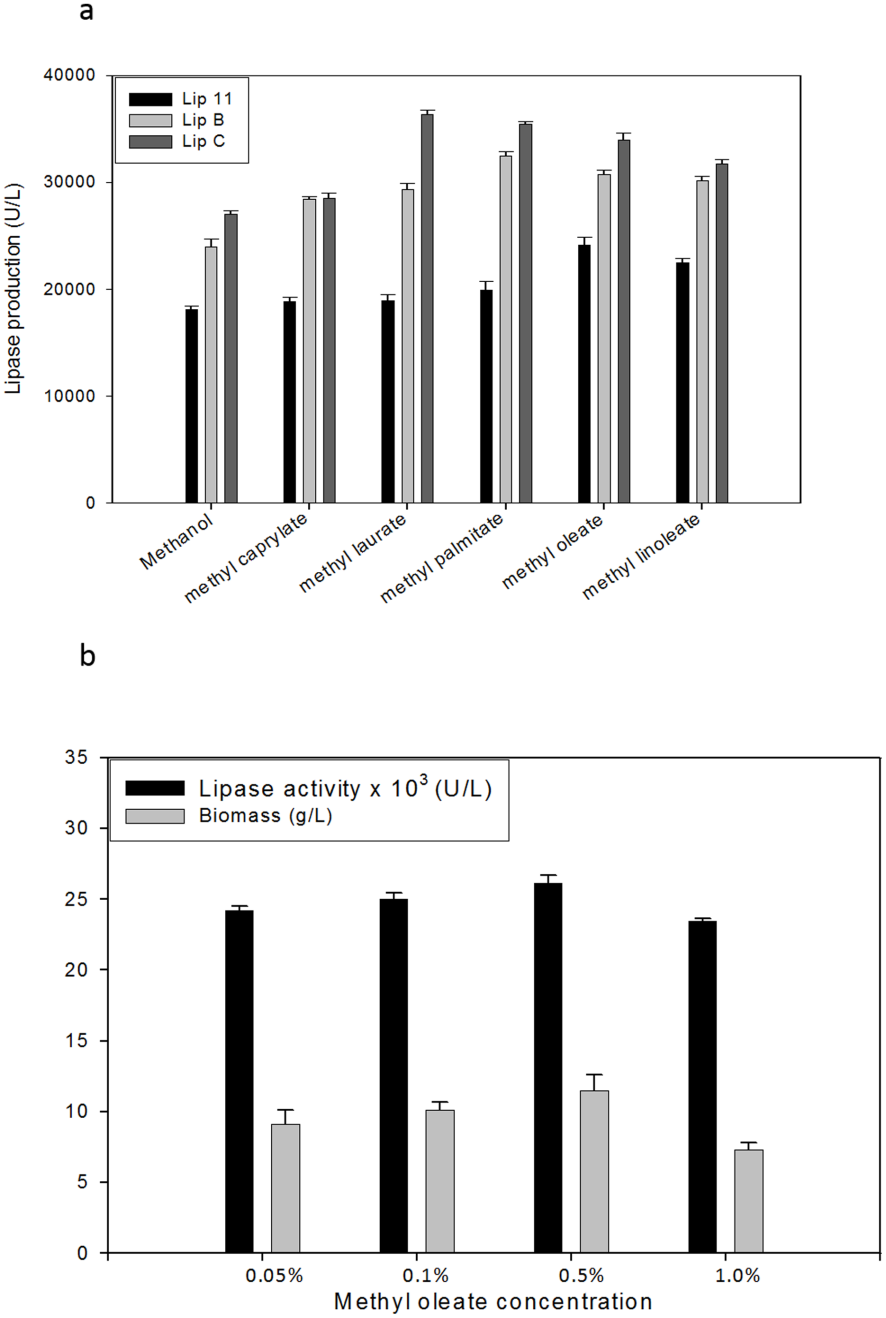
Effect of different methyl esters as an inducer of *AOX*I promoter on lipase production. (a) Lipase production after 48 h of growth as a function of methanol/methyl esters as inducer. The cultured cells in BMMY media were first induced with 0.5% methanol for 3 h, followed by induction with 0.1% methyl ester after 24 h, and 0.5% methanol induction after 24 h as control. Lipase yield was calculated after 48 h of culturing. (b) Methyl oleate concentration optimization.

We screened various methyl esters (0.1%) for their role in lipase over-production. We found that the production was directly dependent on substrate preference of the lipases (figure 3a, S1c, S1b,). The highest production of Lip 11 was achieved by methyl oleate (24160 U/L), followed by methyl linoleate (22491.0 U/L) that was 1.30 fold and 1.24 fold higher than 2% methanol, respectively. Lip A showed maximum production by methyl palmitate (32492 U/L) followed by methyl oleate (30719 U/L) that was 1.35 fold and 1.27 fold higher than 2% methanol, respectively. In contrast, after 48 h, Lip C has maximum production by methyl laurate (36347 U/L) followed by methyl palmitate (35437 U/L) and methyl oleate (33972 U/L) causing an increase by 1.34 fold, 1.31 fold, and 1.25 fold after 48 h, respectively. Thus, we observed that the lipase production varied with methyl esters depending on the nature of lipase expressed. This is in agreement with substrate specificity of these lipases as they are reported to be mid to long chain specific [5], [6]. As oleic acid and methanol are considered as peroxisomal substrates for *P. pastoris*, we selected methyl oleate for further analysis [7]. The concentration of methyl oleate was standardized using Lip11 and 0.5% (v/v) methyl oleate was selected for further studies (Figure 3b). By using 0.5% methyl oleate, total lipase production in all the three enzymes was found to be 30769 U/L, 37532 U/L, 39866 U/L for Lip11, Lip A and Lip C, respectively. This data was obtained after 120 h indicating that the yield was much higher than methanol fed culture. Likewise, higher production yields and productivity were obtained for all the three lipases in methyl oleate fed cultures, without much change in biomass (Table 1).Thus, higher yields were obtained in all the recombinant lipases after single dose of methyl oleate in comparison to four repeated methanol inductions (Table 1).

**Table 1:** Process parameter comparison.

Condition and parameters		Inducers	
		MeOH	Methyl oleate (Batch)
**Temperature/rpm**		30°C/200	30°C/200
**Induction time**		after every 24 h till 96 h	0.5% at 24 h only
**Lipase production (U/L) (120 h)**	**Lip C**	32,866.0±111.1	39,866.0±108.7
	**Lip A**	28,871.0±126.6	37,532.0±78.3
	**Lip 11**	21978.0±121.3	30,769.0±96
**Lipase yield (U/L x^−1^)**	**Lip C**	2753.0±32.4	2870.0±11.6
	**LIP A**	2387.3±12.7	2412.5±21.4
	**Lip 11**	1708.4±21.4	2157.2±33.2
**Productivity (U/L/h)**	**Lip C**	273.8±2.3	332.2±0.9
	**Lip A**	240.6.9±3.5	312.7±4.2
	**Lip 11**	183.2±3.3	256.4±5.4
**Biomass (g/L) dry cell weight**		10.1±0.2	11.2±0.1

First induction was given with 0.5% methanol after culturing the cell in BMMY media for 3 h.

These results indicate that methyl ester may serve as a slow release methanol source in lipase expressing recombinant *P. pastoris*.

### Validating the proposed strategy

**Figure 4:**
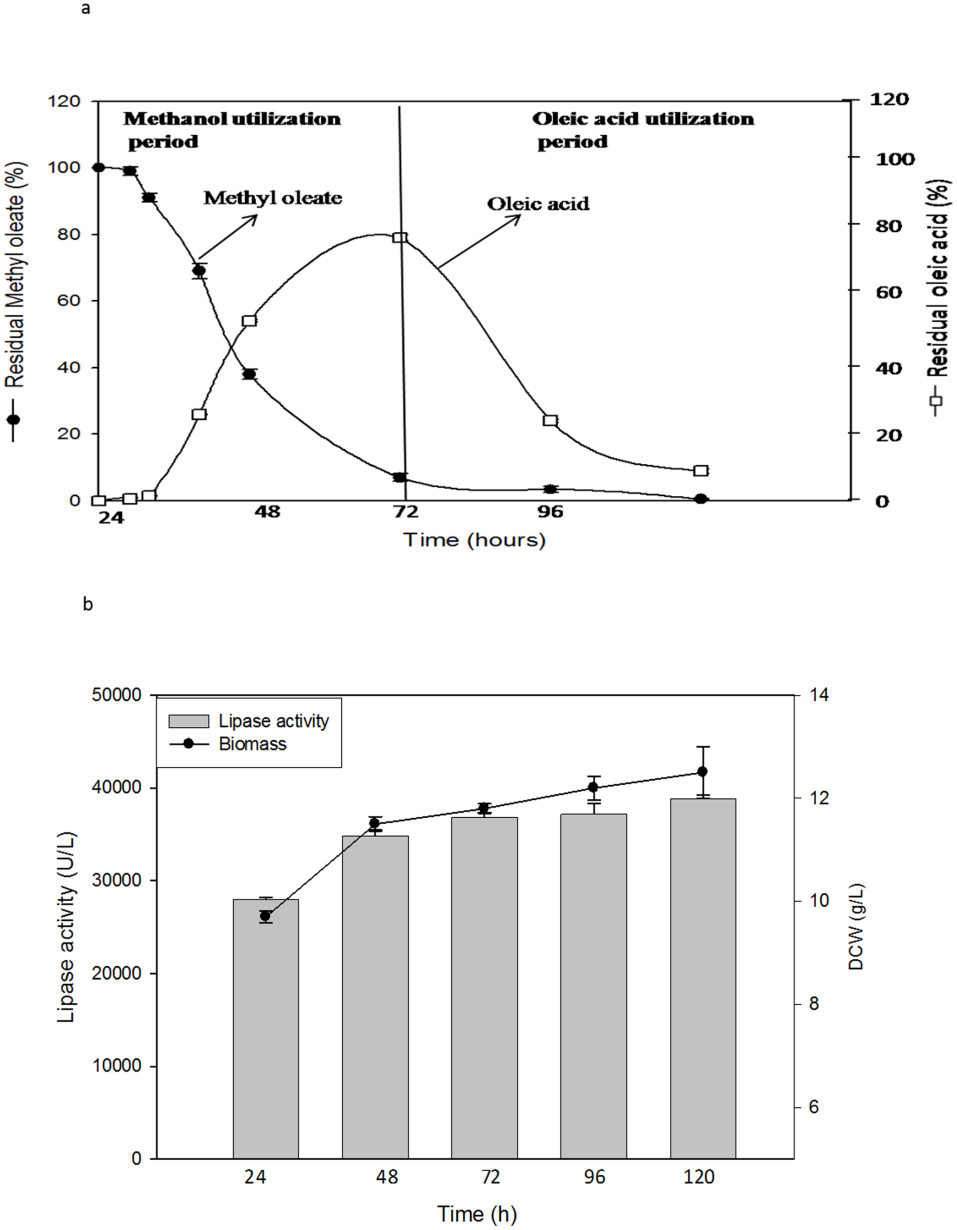
Residual methyl oleate and oleic acid during growth of methyl oleate induced culture of recombinant *P. pastoris* X33 for a period of 120 h (A) and *P. pastoris* cell growth vs incubation time in methyl oleate fed recombinants (B). Concentration of methyl oleate and oleic acid were monitored by gas chromatography and their residual concentration was calculated from peak area, where 0.5% or 17 mM methyl oleate corresponds to peak area 183942 mm^2^ and an equimolar amount of oleic acid corresponds to 172672 mm^2^ as 100%. GC chromatogram is shown in Figure S2.

We validated our proposed strategy by testing if the methyl ester releases methanol slowly that subsequently drives lipase expression. The consumption of methyl oleate and release of oleic acid was monitored by gas chromatography (GC). We have analyzed all the recombinant strains, however only Lip C results are reported in this manuscript (Figure 4a, S2). We found that there was a rapid break down of methyl oleate after 6 h of induction reaching maximum consumption till 72 h of cell culture, with concomitant accumulation of oleic acid. Interestingly, oleic acid was consumed only after 72 h of cell culture. This suggests that methanol, the hydrolytic product of methyl oleate, was initially utilized as an inducer for *AOX*1 promoter as well as carbon source till 72 h. This was followed by rapid utilization of oleic acid till 120 h accompanied by consistence increase in biomass and lipase yield (1.04 fold) (Figure 4a, 4b). From these observations, we inferred that the time span of 120 h could be clearly divided into two phases: (1) methanol utilizing phase (methylotrophy) up to 72 h, where methanol acts as inducer and carbon source simultaneously, (2) fatty acid utilizing phase (fatty acid trophy), where fatty acid serves only as energy source for biomass maintenance when methanol become non repressible and here methanol acts only as inducer. Our results also suggest that *P. pastoris* preferentially utilizes methanol over fatty acid for biomass maintenance.

**Figure 5:**
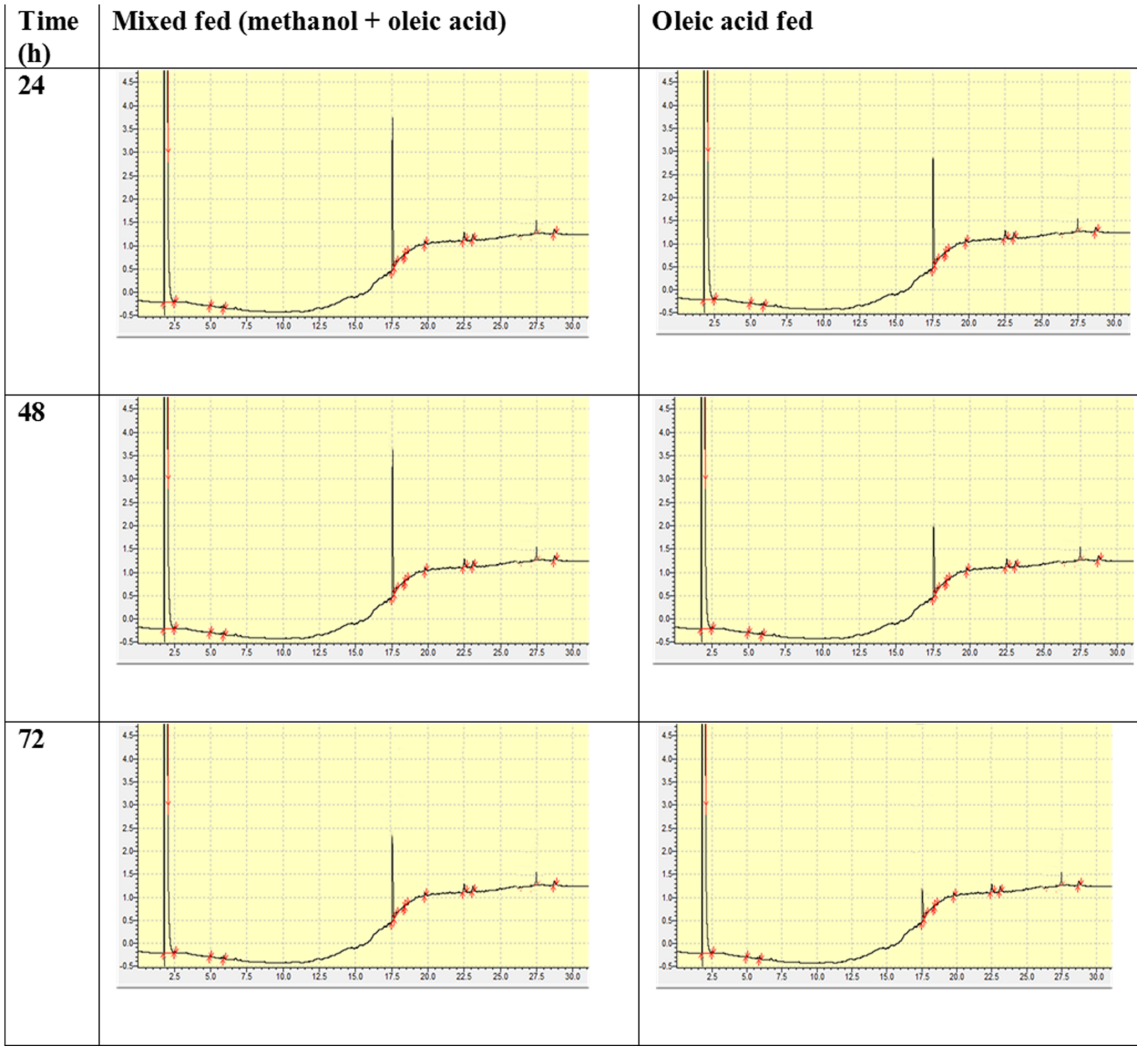
GC chromatogram showing utilization of oleic acid in presence and absence of methanol over a period of 72 h. Peak area at 17.5 min corresponds to residual oleic acid in the medium.

To confirm whether the oleic acid could be used in presence of methanol, we studied the consumption of oleic acid by GC in a mixed fed culture. We additionally introduced 0.1% oleic acid to the methanol (2%) fed culture and compared with culture grown in presence of oleic acid only (Figure 5). We found that oleic acid consumption was suppressed by high amount of methanol concentration (2%) and when the methanol concentration reached below the threshold, the cells utilized oleic acid. However, the consumption started immediately in oleic acid fed cultures.

### Cellular adaptability of recombinant *P. pastoris* during methylotrophy and fatty acid trophy under different culture conditions

**Figure 6:**
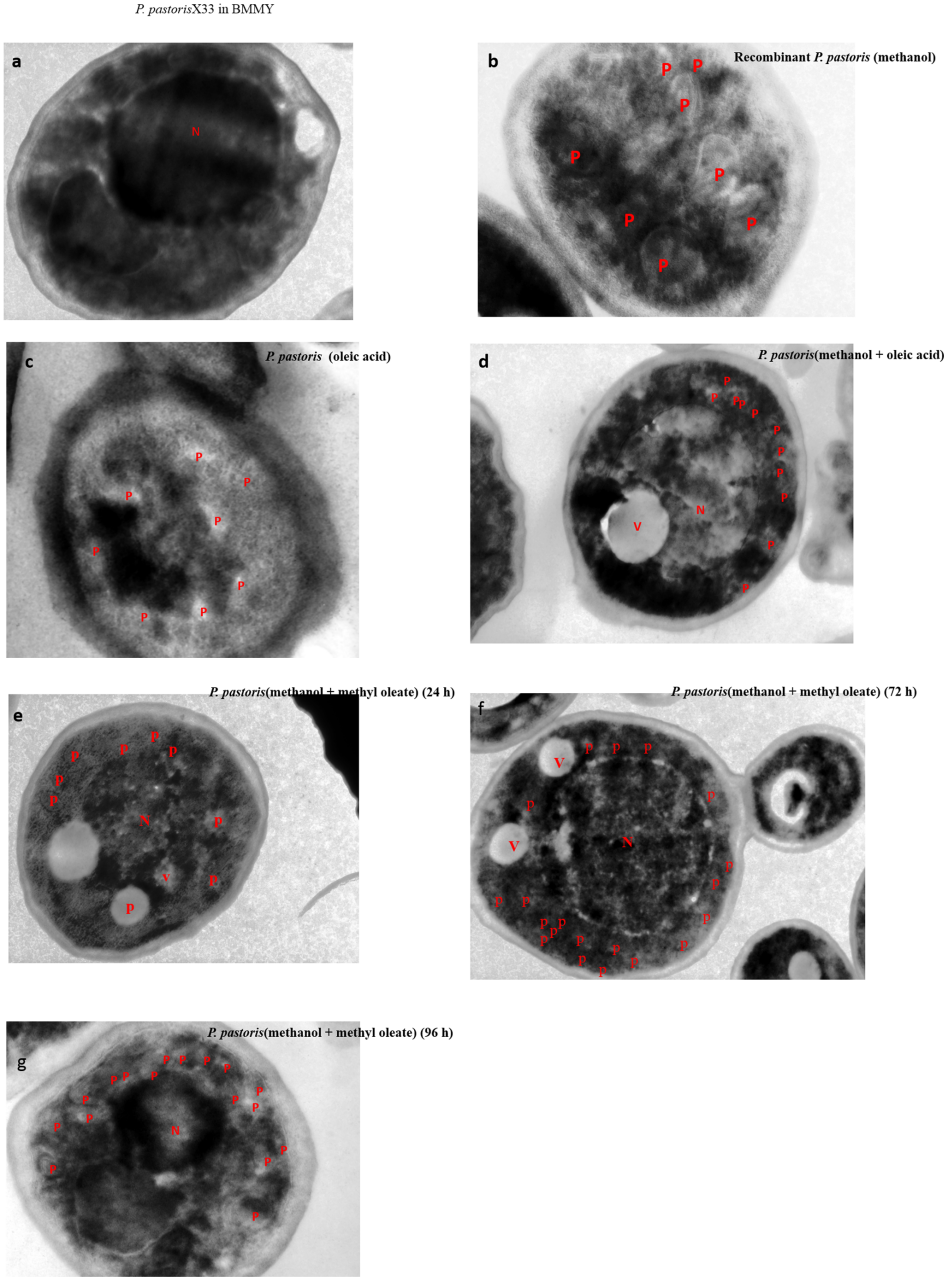
TEM analyses of recombinant P. pastoris under different physiological conditions showing differential peroxisome proliferation. a) Control in BMMY medium 48 h, no peroxisome is visible; b) methanol fed culture 48 h, larger peroxisomes were observed; c) oleic acid fed culture 48 h, smaller and numerous peroxisomes were present; d) mixed fed culture (methanol + oleic acid) 48 h, peroxisome of varying size were observed; e) methyl oleate fed cultures after 24 h, larger peroxisomes few in number; f) methyl oleate fed cultures after 72 h, peroxisomes of varying size were observed, g) methyl oleate fed cultures after 96 h, numerous peroxisomes of varying size were observed. The magnification is 1 µm for all images. N  =  nucleus, V =  vacuole and P =  peroxisome.

There are several reports suggesting the role and function of peroxisomes in methanol metabolism [7], [8]. We performed TEM analysis to further understand the effect of the peroxisomal substrates, methanol and oleic acid, on the physiology of *P. pastoris* X33. We monitored the proliferation of peroxisomes under different culture conditions. *P. pastoris* grown in BMMY was used as a control (Figure 6a) that was devoid of peroxisomes. We found that larger peroxisomes appear when recombinant *P.pastoris* X33 shifted to methanol suggesting their direct role in methanol metabolism (Figure 6b). This is in agreement with previous studies, showing that the membrane bound organelle has a direct role in methanol metabolism; it can intoxicate the cell from the anti-oxidative response that occurrs due to methanol metabolism [4], [7]. According to Yurimoto et al., [9] peroxisomes perform intoxication reaction by two pathways namely: assimilation and dissimilation. During the assimilation pathway, methanol is directly assimilated by the proteins present in the matrix of the peroxisome. After assimilation, it provides energy in the form of ATP used for cell proliferation. At this stage, the cells have large scattered peroxisomes in the cytoplasm due to the presence of matrix proteins. In dissimilatory pathways, fatty acids like oleic acid are consumed in the β– oxidation pathways. Peroxisomes are small in size and mainly rich in enzymes involved in β– oxidation pathways. Similar results were found in the present study where recombinant strains have small and scattered peroxisomes when grown in oleic acid alone (Figure 6c). Similar variations in size and number of peroxisomes were observed during lipase expression in the presence of methyl oleate. Figure 6d shows that in early hours of methyl oleate induction, cells had larger peroxisomes as in methanol supplemented condition and after 72 h, smaller and large number of peroxisomes were observed as in oleic acid grown cells (figure 6e). This clearly supports that lipase expressing *P. pastoris* when grown on methyl esters shifts to two phases of growth: methylotrophy and fatty acid trophy.

## Conclusions

In this study, a method was developed for lipase expressing *P. pastoris* to alleviate repeated methanol feeding problems. It has been clearly shown that methyl oleate can be used as slow release methanol source for the over production of lipase. The results can be summarized as follows:

- There was sustained production of lipase after single dose of methyl oleate in contrast to methanol fed culture that required induction after every 24 h.
- Fatty acid utilization and peroxisome proliferation after 72 h clearly indicated that strain was initially dependent on methanol and later shifted to fatty acid as energy source.
- On the basis of above results, fed batch strategy for methyl oleate can also be developed. So, this is an attractive strategy for over production of lipases in *P. pastoris*.

## Supporting Information

Figure S1 **SDS-PAGE analysis of Lip11 (A) and SDS-PAGE analysis of TALipA and TALipC (B).** 30 µl of crude cell free supernatant was loaded on the 10% SDS-PAGE.
(TIF)

Figure S2 **GC chromatogram.** a. After 3 h induction of methyl oleate (retention time of methyl oleate = 27.5 min, oleic acid = 17.5 min), b. After 24 h of induction of methyl oleate or 48 h of cell culture, c. After 48 h of methyl oleate induction or 72 h of cell culture.
(TIF)
