# Supplementary figures and images for: Novel Strategy of Using Methyl Esters as Slow Release Methanol Source during Lipase Expression by mut+ Pichia pastoris X33
Source: PLoS One. 2014 Aug 29;9(8):e104272. doi: 10.1371/journal.pone.0104272 (PMC4149357; doi:10.1371/journal.pone.0104272)

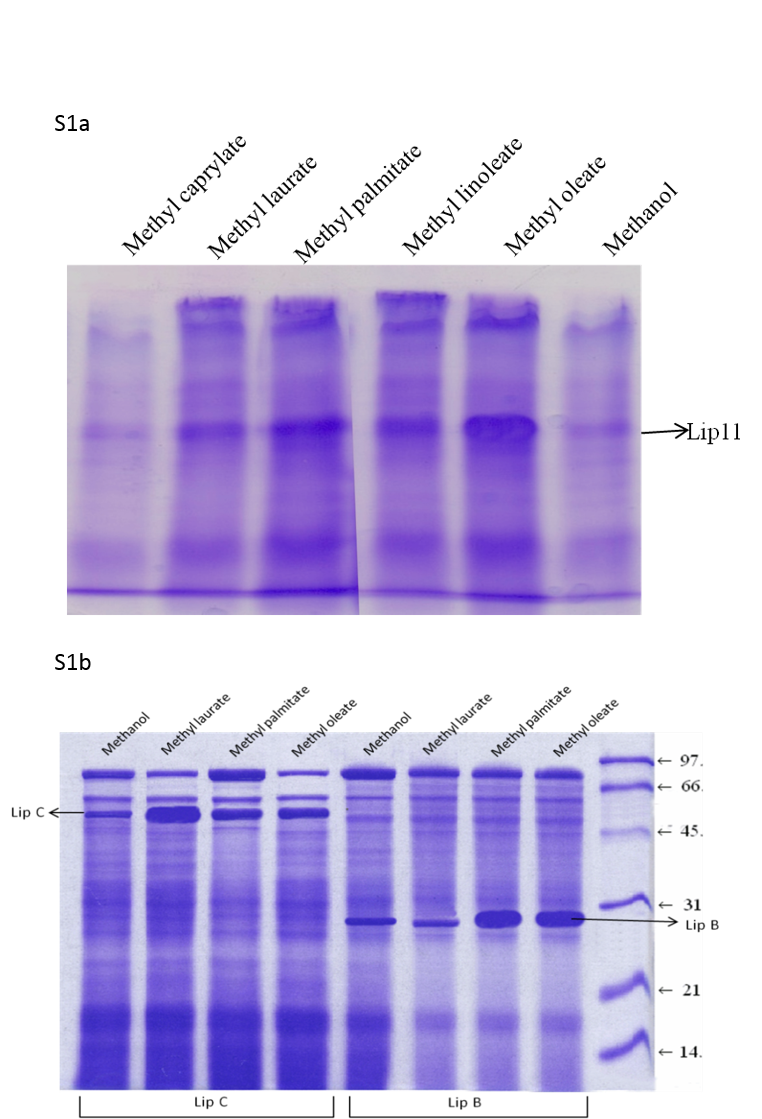

Supplement: Figure S1 — SDS-PAGE analysis of Lip11 (A) and SDS-PAGE analysis of TALipA and TALipC (B). 30 µl of crude cell free supernatant was loaded on the 10% SDS-PAGE. (TIF) [file pone.0104272.s001.tif]

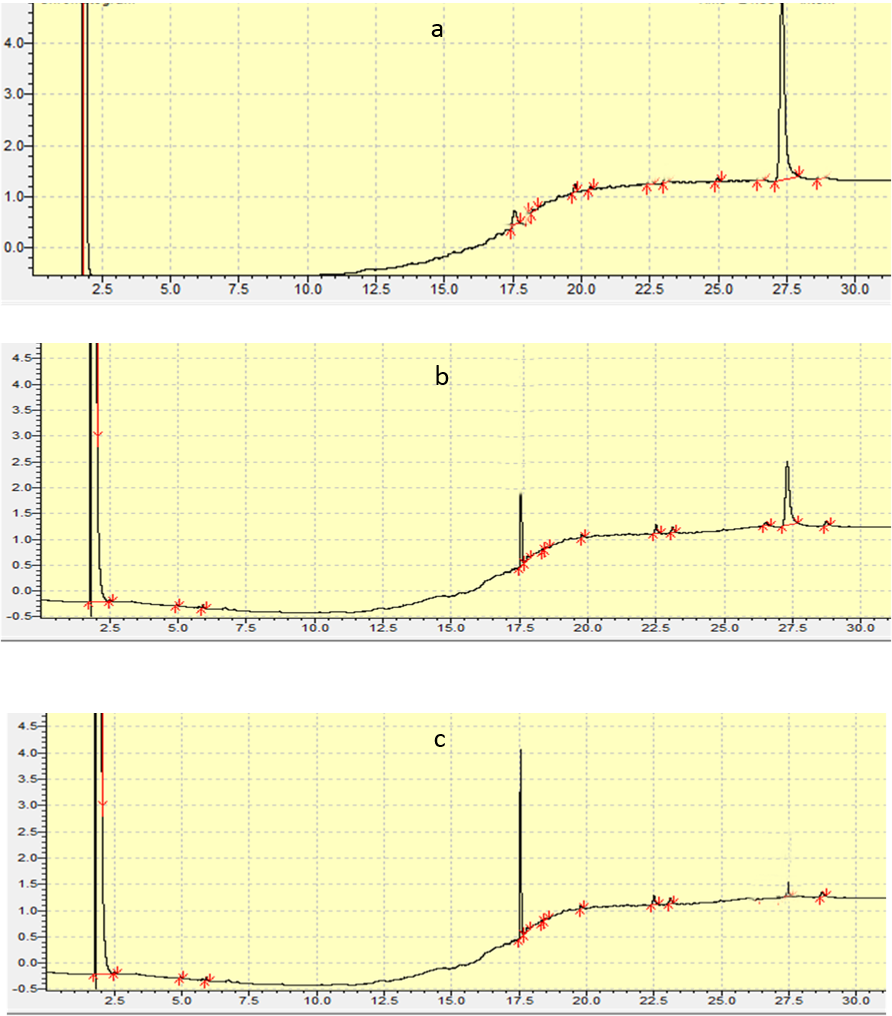

Supplement: Figure S2 — GC chromatogram. a. After 3 h induction of methyl oleate (retention time of methyl oleate = 27.5 min, oleic acid = 17.5 min), b. After 24 h of induction of methyl oleate or 48 h of cell culture, c. After 48 h of methyl oleate induction or 72 h of cell culture. (TIF) [file pone.0104272.s002.tif]
